# Chemical Examination of the Mineral Water of Cooper’s Well, near Raymond, Mississippi

**Published:** 1852-07

**Authors:** J. Lawrence Smith

**Affiliations:** Professor of Chemistry in the University of Louisiana


### Art. IV.—*Chemical Examination of the Mineral Water of Cooper's Well, near Raymond, Mississippi.* By J. Lawrence Smith, M. D., Professor of Chemistry in the University of Louisiana.

The water which forms the subject of this report, is one which has enjoyed a high reputation for medical virtues during several years, it being more especially beneficial in chronic diseases of the intestines.

The water analyzed, was collected in the month of December, 1851, and part of the examination was made at the source.

To most persons who visit this watering-place, its discovery is associated with some curious dreams of the remarkable individual whose name it bears, but its medical virtues remained unknown for sometime after its discovery, and were first ascertained by persons living in its neighborhood. The water is derived from a well dug to the depth of 107 feet in a porous sand rock, which is covered in some places with a coarse conglomerate of pebbles, consolidated by oxide of iron. The depth of the water in the well seldom exceeds five feet; it is said to flow in at the bottom from three different sources, and it will be well to examine their general characters separately, so soon as the water from them can be obtained. It flows most abundantly in winter, and therefore the summer water might be expected to contain more mineral matter. The country around is broken, remarkably dry, enjoying a reputation for general salubrity, and its convenient proximity to the Vicksburg and Jackson railroad is calculated to make it the first watering-place in the South-Western country, either as a resort for invalids or pleasure seekers.

*Chemical Examination of Cooper's Well Water.*—Temperature, 64° Fahr., the air being at 53° Fahr.

Taste—Not unpleasant; slightly mineral.

Odor—Little or no odor, although at times, it is said to have distinctly that of sulphuretted hydrogen.

Color—Transparent, with small yellow flakes floating in it.

Specific gravity, .................. 1,00,147.

Gas, contained in one gallon,

Composed of oxygen, .................. 20

" " nitrogen, .................. 68

" " carbonic acid,—trace;

" " sulphurretted hydrogen,—trace.

Substances contained in one gallon, are,

| | | |
|---|---|---|
| Sulphate of soda, | 11.705 | grains. |
| " " magnesia, | 23.280 | " |
| " " lime, | 42.122 | " |
| " " potash, | .608 | " |
| " " alumina, | 6.100 | " |
| Chloride of sodium, | 4.260 | " |
| " " calcium, | 4.025 | " |
| " " magnesium, | 3.480 | " |
| Silica, | 1.801 | " |
| Peroxide of iron, | 3.362 | " |
| Crenate of lime, | .311 | " |

The deposit which collects from concentrating the water, or after it has stood for a time, contains in 100 grains:

| | |
|---|---|
| Crenate of lime, | 2. |
| Peroxide of iron, | 35. |
| Sulphate of lime, | 25. |
| Water, | 38. |

The iron was found altogether in the yellow particles which float about the water, although it is more than probable that at certain seasons of the year it must be found in the clear water also.

The water when kept loses none of its properties, as has been proved by strict analysis; and at all times, when the effects of the iron are sought for, the sediments should be drank along with the clear water.

The concentrated water appears to lose nothing but a portion of its sulphate of lime. So in that form, when diluted with pure water, similar medical effects might be expected to those produced at the spring; but of course like all other mineral waters, it is only at the source that the patient can expect most advantage; and it is equally true that precautions are to be observed in the use of Cooper's well water.

The diseases to which the water appears mostly adapted, are chronic intestinal diseases, which embrace a class too numerous to be mentioned in this report. It has also been found eminently useful in dropsical affections, as well as in inflammation of the bladder. In the short space of twelve or fifteen years, it is impossible to discover all the diseases to which a water of this description is adapted, as chemical analysis alone cannot guide us in making its application. The remarkable virtues of this water, in the character of diseases before alluded to, are known and acknowledged by several of the most eminent physicians of New Orleans, Mississippi and Alabama.

